# Emotion Regulation as a Transdiagnostic Link Between ADHD and Depression Symptoms: Evidence from a Network Analysis of Youth in the ABCD Study

**DOI:** 10.21203/rs.3.rs-6823726/v1

**Published:** 2025-06-18

**Authors:** Jessica B. Tharaud, Molly A. Nikolas

**Affiliations:** 1 University of Iowa, Department of Psychological and Brain Sciences, Iowa City, Iowa, United States of America

**Keywords:** ADHD, depression, emotion regulation, adolescence, network analysis, developmental psychopathology

## Abstract

**Background:**

Childhood ADHD is associated with greater risk of depression in adolescence and adulthood, with emotion regulation (ER) identified as a potential mediator. However, it remains unclear how distinct domains of ER differentially relate to ADHD and depression symptoms in early adolescence.

**Methods:**

The current analysis estimated a network model using longitudinal, parent-reported data from the Adolescence Brain and Cognitive Development (ABCD) Study 5.1 Data Release in 2023 (*n* = 4,460 complete cases). Nodes were item-level ADHD symptoms averaged across ages 9–12, ER domains (Catastrophize, Distracted, Attuned, and Negative Secondary Emotions) at ages 12–13, and item-level depression symptoms at ages 13–14. We also examined differences in network structure and connectivity by sex, history of ADHD diagnosis, and ADHD polygenic risk score (PRS).

**Results:**

Catastrophize and Distracted were the most important ER bridges between earlier ADHD and later depression symptoms. Two distinct pathways emerged: inattentive ADHD symptoms were linked to depression symptoms (poor eating, feeling worthless) via the Distracted ER dimension, while hyperactive-impulsive ADHD symptoms were linked to depressed mood and anhedonia via the Catastrophize ER dimension. Exploratory network comparisons found similar networks by sex, structural differences by history of ADHD diagnosis, and differences in structure and connectivity by ADHD PRS.

**Conclusions:**

Multiple pathways from ADHD in childhood to depression in early adolescence appear to involve ER difficulty through catastrophizing and distraction when upset. A denser, more interconnected network of symptoms was found among youth with higher genetic risk for ADHD.

## Background

Attention-deficit/hyperactivity disorder (ADHD) is a commonly diagnosed neurodevelopmental disorder with primary symptoms of inattentiveness and/or hyperactivity-impulsivity. Children with ADHD are at greater risk of developing depression in adolescence and early adulthood [1, 2], and one in four U.S. adolescents with ADHD between the ages of 12 and 17 have also been diagnosed with a depressive disorder [3]. The course of depression tends to be more severe and impairing among individuals with ADHD, with earlier onset, greater recurrence, and poorer outcomes, particularly among girls and women [4, 5]. Because of the high overlap between ADHD and depression observed at the genetic level [6], identifying transdiagnostic constructs across development may be particularly useful to uncovering etiological mechanisms linking ADHD and depression.

One such transdiagnostic construct is emotion regulation (ER), the ability to recognize and modulate emotional responses and engage in adaptive behaviors to achieve desired goals [7]. Problems with ER have been associated with ADHD in both children [8] and adults [9] and prospectively with the onset of depression in adolescence [10]. Prior research also points to ER as a potential mechanism explaining the association between ADHD and depression. For example, a cross-sectional study found that both parent- and youth-reported ER fully mediated the link between ADHD diagnosis and depressive symptoms in early adolescence [11]. Similarly, ER partially mediated the link between earlier ADHD symptoms and later depressive symptoms in a prospective longitudinal study of youth aged 9 to 12, controlling for earlier depressive and conduct symptoms [12]. Hyperactive-impulsive symptoms more strongly predicted later ER than inattentive symptoms, suggesting that these core symptom dimensions may be differentially linked to ER. A large, population-based study in the UK found that ER also mediated the *within-person* longitudinal association between ADHD and internalizing symptoms in young children, suggesting ER interventions could be effective at preventing depression and anxiety symptoms in children with ADHD [13]. That shared genetic factors have been found to account for the relationship between ADHD and ER but not for depression ER further indicates that ER may be an effective treatment target [14].

However, the use of composite measures for ER and ADHD symptoms presents a challenge to translating these findings into developing effective interventions. ER is a multidimensional construct that includes distinct domains such as the identification of emotions, awareness of one’s own emotions, access to and implementation of strategies to modulate emotional responses, and ability to maintain self-control when emotionally aroused [7]. Though some research indicates that psychosocial interventions for ADHD may be effective in improving depression symptoms for youth with both ADHD and ER problems [15], it remains unclear how different domains of ER may relate to ADHD and depression symptoms.

Network approaches to psychopathology may be particularly useful for conceptualizing unique relationships between individual symptoms and symptom domains within a dynamic system [16]. From this perspective, symptoms of ADHD, ER, and depression may influence each other across development. Composite scores typically assume the unidimensionality of constructs they measure, limiting inferences about the relationships between symptoms and how these relationships might change over time. Instead, network analysis offers a powerful methodology to describe relationships between symptoms as well as to identify central symptoms that may be ideal treatment targets to prevent down-stream activation of other symptoms [17]. In this way, network modeling of ADHD and depression symptoms with ER domains can identify which ER domains may be most promising treatment targets to prevent the development of depression symptoms among youth with ADHD in adolescence and adulthood.

A challenge to implementing a network model of youth ER is that ER measures created for adults may not necessarily be valid for youth. One of the most widely used measures of ER, the Difficulties with Emotion Regulation Scale (DERS), is a 36-item self-report measure developed for adults [7]. The DERS assesses global, trait-like ER abilities based on a clinical conceptualization across several dimensions: emotional awareness and understanding, engaging in goal-directed instead of impulsive behavior when upset, and access to strategies to regulate emotions [7]. In part due to concerns about the validity of child and adolescent self-report, the scale was adapted for parent-report, and factor analytic work among youth with and without ADHD derived four distinct ER domains: Catastrophize, Negative Secondary Emotions, Attuned, and Distracted. Catastrophize reflects emotional overwhelm and loss of control when upset; Negative Secondary Emotions reflects interference of other emotions such as embarrassment and shame when upset; Attuned refers to lack of awareness and clarity regarding one’s emotions; and Distracted refers to difficulties with concentration, focus, and goal-directed behavior when upset. Among adolescents with ADHD, Catastrophize and Negative Secondary Emotions demonstrated incremental validity in predicting adolescent self-reported and parent-reported depression symptoms beyond the adolescent self-report DERS [18]. We chose to use the four ER domains as network nodes instead of individual items to reduce overlap between items of similar wording and to examine broader domains of ER.

The goal of the current study was to identify which ER domain(s) most strongly link symptoms of ADHD and depression in a network model spanning late childhood to early adolescence in the ABCD cohort. In accordance with a mediational model of ER, we used parent-reported symptoms of ADHD in childhood, ER domains in late childhood, and depression symptoms in early adolescence. Available depression measures in the ABCD study provided more comprehensive coverage of symptoms for parent-report than youth self-report (i.e., seven parent-report symptoms vs. three self-report symptoms). Therefore, we used parent-report of depression even though some research suggests that adolescents may have better insight into their own depression symptoms than parents [19].

Based on prior work [18], we hypothesized that Catastrophize and Negative Secondary Emotions would be the most important ER domains linking ADHD and depression symptoms, followed in descending order by Distracted and lastly Attuned. Additional evidence has indicated that trajectories of ER and depression symptom development across childhood and adolescence differs by both sex and ADHD diagnosis [20] and that ER may more strongly mediate ADHD and depression for young adult women than men [21]. Furthermore, higher genetic risk for ADHD as measured by a polygenic risk score (PRS) has been associated with greater odds of ADHD diagnosis, higher symptom severity, and greater odds of co-occurring psychopathology such as depression as well as ER problems [22]. These findings suggest that relationships between ER and ADHD and depression symptoms might differ based on sex, ADHD diagnosis, and genetic risk for ADHD. As such, we conducted exploratory network comparison analyses compare network structure and connectivity by sex, ADHD diagnosis, and ADHD PRS.

## Methods

### Participants

Data were drawn from the Adolescent Brain and Cognitive Development (ABCD) Study, the largest longitudinal study of child brain development in the U.S. [23]. The sample of 11,868 youth was selected to be diverse and nationally representative [24]. Baseline data were collected when youth were 9 to 10 years old. Four years of annual follow-up data had been released at the time of data analysis (Data Release 5.1), but only a subset of participants had available data at year 4 (*n* = 4,754), when depression data were measured for the current analysis. Most participants (*n* = 11,866; 99.9%) had at least one data point across time points available for analysis, and 4,460 participants (93.8% of participants with available depression symptoms at year 4; 37.6% of total sample) had complete data including ADHD at baseline, year 1, and/or year 2, ER at year 3, and depression measures at year 4. Previous research using ABCD data has identified caregiver education level, employment, and preference for Spanish over English as predictors of missed visits and study withdrawal [25]. Caregivers provided written consent and youth provided verbal assent. Procedures were overseen by local IRBs at each study site.

### Measures

#### Demographics.

Caregivers reported on demographic characteristics such as youth sex, race and ethnicity, and family income at baseline (see Table 1).

#### Difficulties in Emotion Regulation Scale – Parent Report (DERS-P).

Caregivers reported their perceptions of adolescent ER difficulties at ages 12 to 13 (follow-up year 3) using the DERS-P [18], a parent-report adaptation of the widely used Difficulties with Emotion Regulation Scale (DERS) [7]. Among adolescents with and without ADHD, the DERS-P demonstrated incremental validity over the DERS in ADHD and internalizing symptoms as well as similar internal consistency and concurrent and concurrent validity [18]. Each of the 29 items are rated from *1* (*almost never*) to *5* (*almost always*). Two DERS-P items (“difficulty concentrating” and the belief “that he/she will end up feeling very depressed” when upset) conceptually overlapped with ADHD and depression items on the CBCL and were omitted from analyses. Eight items were reverse-coded (six items from the Attuned subscale and two from the Catastrophize subscale). Items in each subscale were summed and scaled, with higher scores reflecting greater ER difficulties. Internal consistency in the current study was good for all four subscales before (Cronbach’s α ranges from .91 to .92; ω_total_ ranges from .89 to .94) and after removal of the two items (Cronbach’s α ranges from .88 to .92; ω_total_ ranges from .88 to .93).

#### ASEBA Child Behavior Checklist 6–18 (CBCL).

Caregivers’ report of youth behavioral problems was measured yearly using the CBCL [26]. The DSM-5 Oriented scales consist of seven ADHD and thirteen depression items rated on a three-point Likert scale (*0* = *not true*; *1* = *sometimes true*; *2* = *often true*). Both scales are normed by age and sex for comparison to clinical population norms. ADHD items were averaged across baseline and follow-up years 1 and 2 (ages 9 to 12) for maximum sample size and data inclusion. Depression items were used from follow-up year 4 (ages 13 to 14). Internal consistency was acceptable for both the ADHD scale (Cronbach’s α ranged from .84 to .85; ω_total_ = .89 for all timepoints) and the depression scale (Cronbach’s α = .82; ω_total_ = .85).

#### Kiddie Schedule for Affective Disorders and Schizophrenia Computerized (KSADS-COMP).

The KSADS-COMP is a computerized semi-structured clinical interview to diagnose psychiatric disorders in school-aged children [27]. Parents completed the KSADS-COMP yearly across all timepoints. Youth who met current or past criteria for ADHD or reported history of an ADHD diagnosis at any point during the study were considered to have a positive history of ADHD diagnosis. We decided to be inclusive given previous evidence of diagnostic instability in ADHD across development [28] and the potential impact of large differences in sample size on network comparisons [29].

#### ADHD Polygenic Risk Score (PRS).

The PRS is a continuous measure of an individual’s genetic risk for a phenotype that aggregates common genetic variants previously identified with the phenotype in large genome-wide association studies (GWAS). We used summary statistics from the largest and most recent ADHD GWAS [6] and individual genotype data to calculate youth ADHD PRS in the current sample. Genotyping was conducted on baseline saliva and blood samples with the Smokescreen Genotyping array at Rutgers University Cell and Data Repository [30]. Data imputation used the TOPMED reference panel. Additional information on genotype collection and processing has been published elsewhere [31]. Standard quality control steps were performed using PRSice-2 software on a Linux operating system [32]. Single nucleotide polymorphisms (SNPs) with an imputation quality score of at least .80 were included, and linkage disequilibrium was estimated using unrelated European individuals. PRSs were residualized on the first ten ancestry principal components to control for population stratification and then scaled. Five *p*-value thresholds were compared (1, .20, .05, .0005, 5e-08) to *t*-scores on the CBCL ADHD scale at baseline. The threshold with the highest *R*^*2*^ value (*p* = .20) in the current sample was selected to calculate the ADHD PRS for each participant.

### Data Analytic Plan

#### Node Selection.

ADHD items (ages 9 to 12), ER domains (ages 12 to 13), and depression items (ages 13 to 14) were selected as nodes in the network. Bivariate Spearman correlations indicated that the strongest associations were observed within communities (e.g., depression items correlated strongly with other depression items). Highly correlated items appeared conceptually distinct and were thus retained in the network. Variances among the selected nodes were below 0.05 for two depression items indicating self-harm and suicidal ideation (“deliberately harms self or attempts suicide” and “talks about killing self”). These two items were omitted from the network analyses because low endorsement can impact network structure [33].

#### Network Estimation and Visualization.

Network models were estimated in *R* version 4.4.0 as Gaussian graphical models via version 1.6 of the *bootnet* package [34]. A regularized network of partial correlations was estimated with the method “cor_auto” to automatically detect ordinal items and maximize data inclusion with pairwise comparisons. Nonzero network edges were subsequently refitted without further penalization, which maximizes the precision of edge estimates without impacting network structure [35]. Networks were visualized using version 1.9.8 of the *qgraph* package [36], with the most central nodes placed in the center of the network [37]. Unless otherwise noted, all centrality values and edge weights are bootstrapped estimates.

#### Accuracy and Stability Checks.

The accuracy and stability of edge estimates and centrality indices were examined using nonparametric bootstraps of 2500 samples. Centrality indices were only interpreted if the centrality stability (CS) coefficient was at least .50 [34].

#### Network Centrality.

Centrality indices quantify the relative importance of each node to the overall network. High centrality indicates that a node has many strong associations (i.e., partial correlations) with other nodes in the network. The expected influence of a node sums edges between that node and all other nodes in the network. Strength centrality sums the *absolute value* of all edges between one node and all other nodes in the network, thus considering both positive *and* negative associations. We descriptively characterized the network model using both expected influence and strength centrality.

#### Bridge Centrality.

The bridge expected influence of a node sums all edges between that node and nodes in *different communities* (i.e., excluding nodes in the same community). We identified communities as ADHD symptoms, ER domains, and depression symptoms. To address our primary research question, we compared bridge expected influence values of DERS-P subscales to identify which ER domain(s) most strongly bridged earlier ADHD and later depression symptoms. Previous research has suggested that bridge symptoms may be most relevant to identifying transdiagnostic processes shared by co-occurring disorders [38].

#### Network Comparisons.

Networks were compared using the *NetworkComparisonTest* package version 2.2.2 [39] in *R* version 4.3.1. The nonparametric NCT function was used with 2500 bootstrapped iterations and False Discovery Rate (FDR) correction for multiple testing. Only participants with complete data were used in analyses to ensure comparability across networks. Correlation matrices were not positive-definite, so nonparanormal transformations were applied using the “cor_method = npn” argument via the *huge* package version 1.3.5 [40]. Visualizations of compared networks were constrained to the same layout with the “averageLayout” function, fixing the edge width to the absolute value of the maximum edge weight.

## Results

### Descriptives

The final sample size consisted of 11,866 youth with at least one data point available for analysis across the five timepoints. The proportion of missing data increased with time: < 0.1% at baseline, 5.6% at year 1, 8.2% at year 2, and 15.4% to 16.5% at year 3. Among youth with available year 4 data, 1.6% of data were missing at year 4. The sample of participants with baseline data and with data at year 4 significantly differed in terms of youth race, parental education, and parental combined income according to chi-squared tests of independence (see Table 2 for descriptive statistics).

### Network Estimation and Visualization

**Fig. 1:**
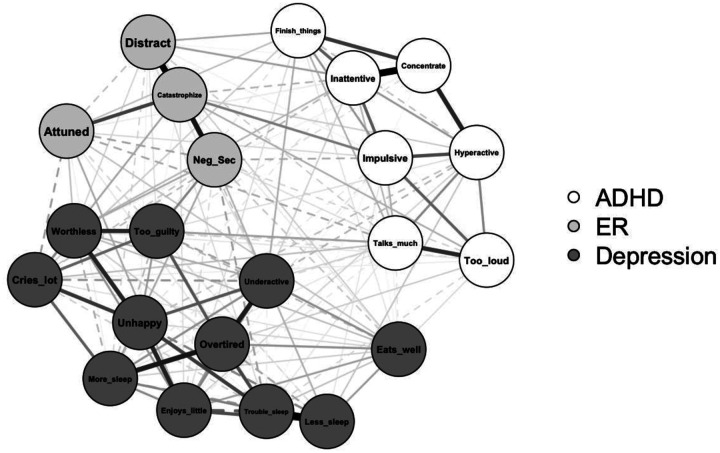
ADHD, Emotion Regulation (ER), and Depression Network Visualization *Note*. Connections between nodes are regularized partial correlations refitted. Edge estimates were refitted without additional penalization after network structure was estimated for precision. Thicker lines reflect stronger partial correlations, and dashed lines indicate negative associations.

**Fig. 2:**
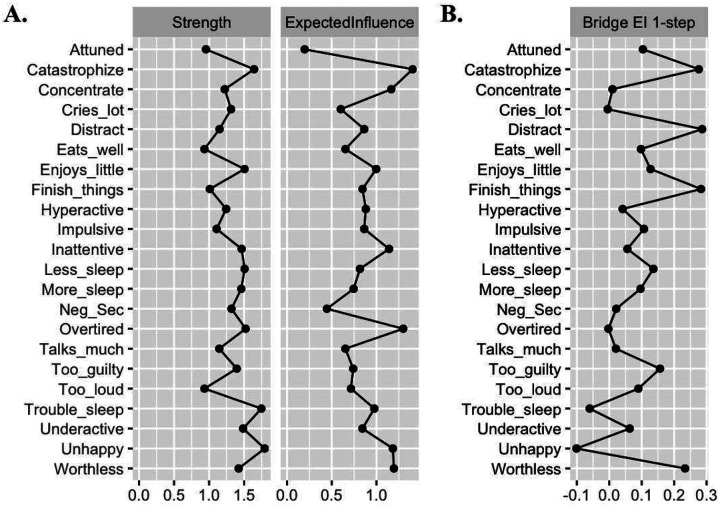
Unstandardized Centrality Values of Network Nodes The figure displays unstandardized strength and expected influence values for each node in the network. DERS-P = Difficulties with Emotion Regulation Scale, DEP = Depression. Strength centrality sums all non-zero correlations. Expected influence sums the absolute value of all non-zero correlations. Bridge expected influence sums the absolute value of all non-zero correlations for nodes in differing communities (i.e., ADHD, ER, and/or depression).

The estimated network (visualized in Figure 1) included 22 nodes: 7 ADHD items, 4 ER subscales, and 11 depression items. Of 231 possible edges, 165 were non-zero (71.4%), indicating a highly dense network. Edge weights ranged from −.20 to .61 (*M* = .04). Visual inspection of the bootstrapped edge-weight estimates suggested acceptable width of confidence intervals (see Figure S1 in supplementary materials). Strength centrality, expected influence, edge weight accuracy, and bridge expected influence indices were sufficiently stable for interpretation, CS-coefficients > .50 (see Figure S2 for visualization; see Table S1 for values). Values of network centrality and bridge centrality indices are plotted in Figure 2; bootstrapped estimates are listed in Table 3. Bootstrapped edge weight estimates (partial correlations) for edges in the network that include DERS-P subscales are listed in Table S2 in supplementary materials.

### Network Centrality

Catastrophize was the node with the highest overall strength centrality in the network and the ER domain with the highest expected influence. Bootstrapped difference tests were significant for both strength centrality and expected influence (estimates = .05) and indicated that Catastrophize was significantly more influential in the overall network than the other three ER domains (see Figure S3 and Figure S4 for visualized comparisons)^1^.

### Bridge Centrality

We examined bridge expected influence to identify which ER dimensions were the strongest bridges between childhood ADHD and adolescent depression symptoms. Distracted had the highest bridge expected influence, followed in descending order by Catastrophize, Attuned, and Negative Secondary Emotions. Bootstrapped difference tests found that bridge expected influence did not significantly differ between the highest subscales (Distracted vs. Catastrophize) or the lowest subscales (Attuned vs. Negative Secondary Emotions) but that all other differences between subscales were significant, *p* < .05 (see Figure S5 for visualization of comparisons).

#### Specific edges and nodes

Catastrophize was uniquely associated with four ADHD items (“can’t sit still, restless, or hyperactive”, “impulsive or acts without thinking”, “inattentive or easily distracted”, “unusually loud”) and five depression items (“there is very little he/she enjoys”, “cries a lot”, “doesn’t eat well”, “underactive, slow-moving, or lacks energy”, “unhappy, sad, or depressed”) and was the only DERS-P subscale positively associated with all three other subscales. The strongest edge between Catastrophize and an ADHD item was “impulsive or acts without thinking” and with a depression item was “cries a lot”. Interestingly, Catastrophize was positively associated with three hyperactive-impulsive symptoms of ADHD and negatively associated with one inattentive symptom of ADHD (“inattentive or easily distracted”). Additionally, Catastrophize was positively associated with several core symptoms of depression (e.g., “little he/she enjoys”, “unhappy, sad, or depressed”).

The Distracted subscale was uniquely associated with five ADHD items and seven depression items. The strongest connection between Distracted and an ADHD item was “inattentive or easily distracted” and with a depression item was “doesn’t eat well”. Associations with ADHD items were positive between three inattentive items (“inattentive or easily distracted”, “can’t concentrate, can’t pay attention for long”, “fails to finish things he/she starts”). Distracted was positively associated with one hyperactive-impulsive ADHD item (“impulsive or acts without thinking”) and negatively associated with one hyperactive-impulsive item (“talks too much”). Though Distracted was associated with the same ADHD items and in the same directions as Attuned, the two subscales were negatively associated.

### Network Comparisons

#### Sex

**Fig. 3:**
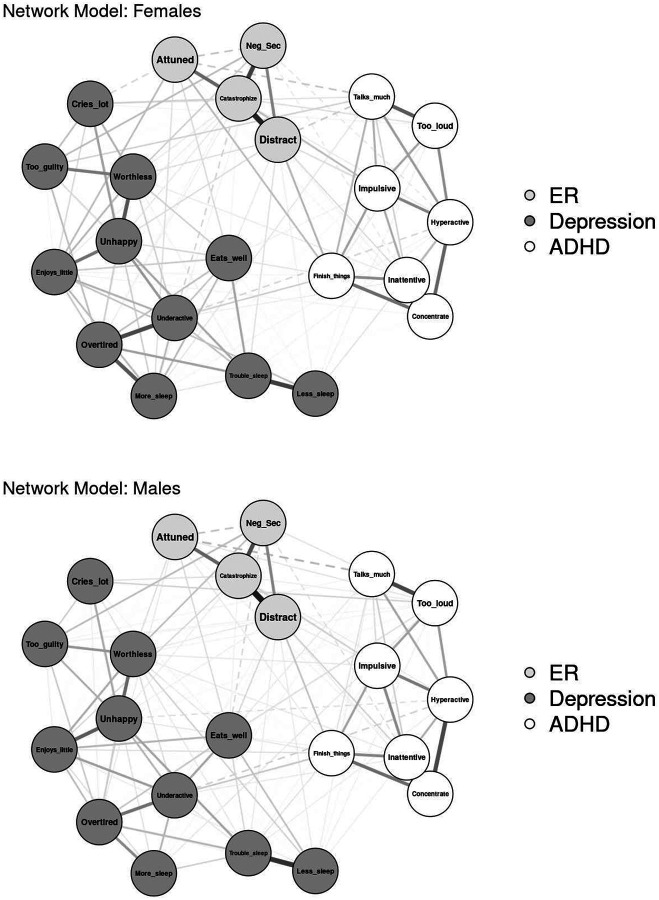
Visualization of Network Comparison by Sex

Networks were modeled separately for youth with complete data who were identified by their parents or caregivers at baseline as female (*n* = 2,104) or male (*n* = 2,355; see Figure 3 for visualization). Three individuals identified as “other” were excluded from analyses. The network of female youth resulted in 126 out of 231 non-zero edges (54.6%), with edge weights ranging from −.08 to .61 (*M* = .04). The network of male youth had 137 out of 231 non-zero edges (59.3%), with edge weights ranging from −.10 to .59 (*M* = .04). Neither the network invariance test (*p* = .696) nor the invariant global strength test (*p* = .307) indicated significant differences in network structure or connectivity. Centrality invariance tests found that “sleeps more than other kids” had significantly greater strength and expected influence in the network of female youth (strength = 0.77; expected influence = 0.76) than in the network of male youth (strength and expected influence = 0.47), *p*s = .047.

#### History of ADHD Diagnosis

**Fig. 4:**
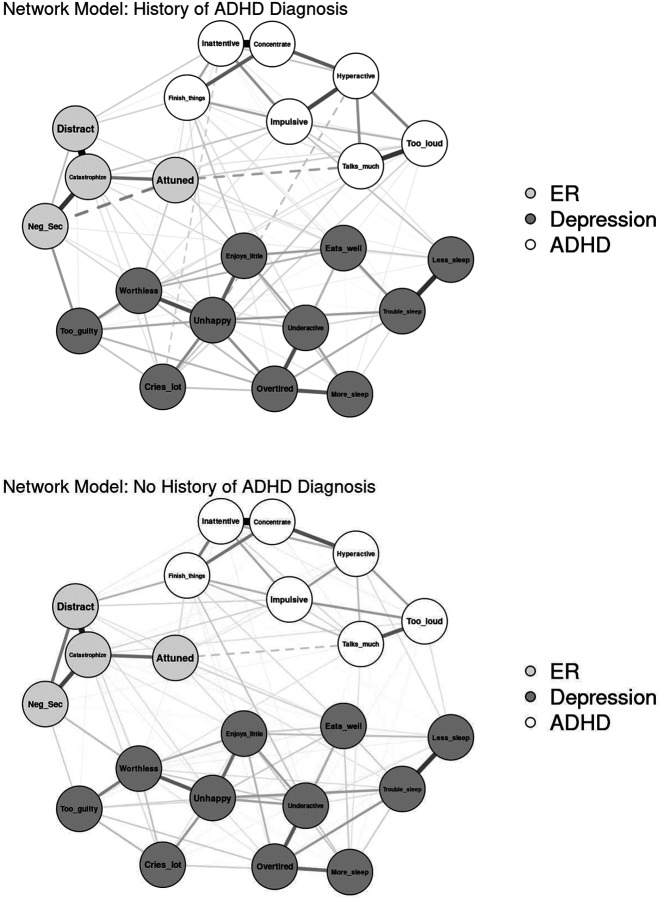
Visualization of Network Comparison by History of ADHD Diagnosis

Networks were compared by whether youth met criteria for current or past ADHD on the parent-reported computerized KSADS (see Figure 4 for visualization). The final sample size consisted of 1,099 youth with current or lifetime ADHD (24.6%) and 3,361 youth with no ADHD history (75.4%). The network of youth with history of ADHD diagnosis resulted in 106 out of 231 non-zero edges (45.9%) that ranged from −.18 to .58 (*M* = .04). The network of youth without history of ADHD had 119 out of 231 non-zero edges (51.5%) that ranged from −.09 to .56 (*M* = .04). The network invariance test found significant differences in network structure between the two networks (*p* = .008). The invariant global strength test was not significant (*p* = .057), indicating similar levels of overall network connectivity. Edge invariance tests found stronger edges in the network of youth with history of ADHD than without history ADHD between “can’t sit still, restless, or hyperactive” and “impulsive or acts without thinking” (ADHD edge = .29; non-ADHD edge = .13) and between Catastrophize and Distracted, *p*s = .046 (ADHD edge = .55; non-ADHD edge = .43). Centrality invariance tests found no significant differences between centrality indices in the ADHD and non-ADHD networks, *p* > .05.

#### ADHD PRS

**Fig. 5:**
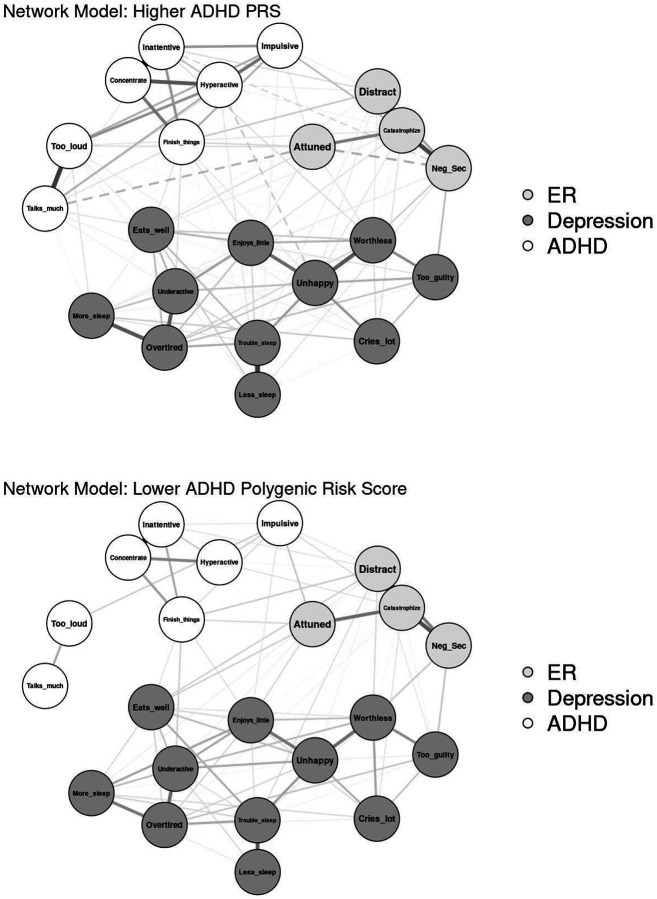
Visualization of Network Comparisons by ADHD Polygenic Risk Score (PRS)

Networks were modeled separately for youth with ADHD PRS above and below the sample median (−0.42; *n* = 1,973 each; see Figure 5 for visualization). The network of youth with higher ADHD PRS resulted in 111 out of 231 non-zero edges (48.1%), with edge weights ranging from −.12 to .61 (*M* = .04). The network of youth with lower ADHD PRS had 82 out of 231 non-zero edges (35.5%), with edge weights ranging from .00 to .49 (*M* = .03). The network invariance test found significantly different network structure, *p* < .001. The global strength invariance test found significantly greater connectivity in the high PRS network (9.77) than the low PRS network (7.45), *p* < .001.

Edge invariance tests found that seven edge weights were significantly greater in the high PRS network: six between pairs of ADHD nodes, and the seventh between Catastrophize and Distracted, *p*s = .013. Centrality invariance tests found significantly greater strength and expected influence of all ADHD items in the high PRS network (all *p*s = .004). Catastrophize had significantly greater strength (*p* = .007) and expected influence (*p* = .004) in the high PRS network than in the low PRS network. Conversely, Attuned had significantly greater expected influence in the low PRS network than in the high PRS network (*p* = .004). In addition, “unusually loud” had significantly greater bridge expected influence (*p* = .016) and “sleeps less than most kids” had significantly greater overall expected influence (*p* = .021) in the high PRS network than the low PRS network. Results for significant edge and centrality invariance tests are listed in Table 4.^2^

## Discussion

To our knowledge, this is the first network analysis to examine ER dimensions as a bridge between childhood ADHD and adolescent depression using a network model. We initially hypothesized that Catastrophize and Negative Secondary Emotions would be the most important connectors between ADHD and depression. We found partial support for our hypotheses: Catastrophize and Distracted (not Negative Secondary Emotions) more strongly bridged between earlier symptoms of ADHD and later symptoms of depression. Connectivity patterns in the network suggested two mediational pathways that may differ for inattention versus hyperactivity-impulsivity symptoms. Exploratory comparisons of the network model found similar networks when compared by sex, some structural differences by history of ADHD diagnosis, and a more strongly interconnected network with structural differences in youth with relatively higher ADHD genetic risk. Overall, findings suggest that Catastrophize and Distracted are the two ER domains that most strongly link ADHD in childhood to later depression symptoms and that these links may be stronger for individuals with higher genetic risk for ADHD.

Contrary to our hypothesis, Distracted was one of the important ER bridges in the network, even though we removed items that clearly overlapped with ADHD symptoms. This finding is consistent with previous network analyses that identified concentration as a common bridge symptom between depression and other psychological disorders [38]. However, Distracted demonstrated fewer unique associations with depression items and was significantly less central to the network overall than Catastrophize. These findings suggest that Catastrophize plays a relatively more influential role in linking ER difficulties to symptoms of ADHD and depression.

The pattern of significant edges in the network point toward somewhat separable pathways to depression involving inattentive versus hyperactive-impulsive symptoms of ADHD. Notably, there appeared to be a double dissociation, such that Catastrophize was positively associated with hyperactive-impulsive symptoms of ADHD but not inattentive symptoms, whereas Distracted was positively associated with inattentive symptoms of ADHD but only one hyperactive-impulsive symptom. This finding suggests that early adolescents with hyperactive and impulsive behavior may be more likely to catastrophize to the worst possible outcomes when upset and be more prone to depressive symptoms such as anhedonia, sadness, and crying. On the other hand, early adolescents with inattention problems may be more likely to be more distracted and less productive when upset, increasing the risk of depressive symptoms such as eating poorly and feeling worthless or inferior. Given that Catastrophize and Distracted were positively associated, one reason for the higher severity and impairment associated with the combined subtype of ADHD may be that both pathways are active and interact, increasing risk of other psychological symptoms. More work is needed to determine the cascading effects of ADHD symptom dimensions on later depression and potential mechanistic differences involving inattention versus hyperactivity-impulsivity.

Additionally, exploratory network comparisons found minimal differences in networks compared by child sex. Network comparisons demonstrated similar network structure and connectivity for male and female adolescents. This finding is somewhat surprising given prior evidence of increasing emotional and depressive symptoms across adolescence for girls but not boys with ADHD [19] and evidence that ER more strongly mediates ADHD and depression for young adult women than men [20]. However, our finding is consistent with another study of younger children that found similar patterns in longitudinal mediation models stratified by sex [13]. One possibility may be that sex differences emerge later in development, particularly given that the early adolescents in the current study may not yet have undergone pubertal changes.

Network comparison by history of ADHD diagnosis indicated similar network connectivity and some differences in network structure, after correction for multiple comparisons. Specifically, the edge between Catastrophize and Distracted and the edge between hyperactive and impulsive ADHD symptoms were significantly stronger in the network of youth with lifetime diagnosis of ADHD compared to those without any ADHD diagnostic history. While stronger connections in the network might have been expected for the youth with history of ADHD diagnosis, one possibility may be that the unequal sample sizes between groups may have reduced our power to detect significant differences. Nevertheless, that the edge between Catastrophize and Distracted remained significant after this relatively conservative approach further emphasizes the importance of both domains of ER in linking symptoms of ADHD and depression.

Network comparisons by ADHD PRS indicated differences in network connectivity and structure. Specifically, the overall network of youth with relatively higher ADHD PRS was significantly stronger and more densely connected. Nearly all ADHD symptoms demonstrated significantly greater edge weights and centrality in the higher ADHD PRS network, underscoring that genetic liability is associated not only with mean levels of ADHD symptoms but also the interconnectedness and strength of their relationships with each other. That this finding did not replicate for the depression symptoms suggests it is specific to ADHD and not generalizable to relationships between depressive symptoms. There were also differences in the most important ER bridges: Catastrophize was significantly more central and the association between Catastrophize and Distracted was significantly stronger in the higher ADHD PRS network than in the lower ADHD PRS network. This finding extends previous work demonstrating that genetic overlap may be primarily responsible for the correlation between ADHD and emotional symptoms across childhood and adolescence [17, 41, 42]. Taken together, these findings support the importance of genetic risk to the co-development of ADHD and ER difficulties across early adolescence.

In addition, the results of this study may have important clinical implications. Bridge symptoms may be particularly relevant to identifying promising mechanisms of the co-occurrence of psychological disorders, which are also prime targets for treatment and preventative interventions [38]. Treating bridge symptoms may prevent the activation of downstream nodes that may lead to co-occurring symptoms. Thus, the current findings indicate that future research should examine the clinical utility of treating catastrophizing and distraction when upset to prevent depressive symptoms among youth with ADHD symptoms.

### Limitations

Interpretations of study results should be made in the context of study limitations. One limitation is the lower sample size of depression symptoms at year 4. This is likely not missing at random as we have assumed due to study dropout and the availability of data dependent on study site. The power to identify significant differences in network comparisons by sex and ADHD diagnosis may have been limited by further reductions in sample size by group. Our decision to control for multiple comparisons may also have led to more conservative estimates [43]. However, the full sample was still quite large.

In addition, we averaged ADHD symptoms across baseline, year 1, and year 2. While this strategy allowed us to maximize the sample size, it is possible that ADHD symptoms at different ages may relate differently to later ER and depression symptoms. Similarly, our analytic strategy did not employ a longitudinal method that could separate temporal from contemporaneous effects or within-person from between-person effects. We also did not control for ER or depression symptoms at earlier timepoints.

## Conclusions

The current study found evidence of two differential pathways from ADHD symptoms to ER difficulties to depression symptoms across a longitudinal network analysis of early adolescents in the ABCD Study. Pathways linked hyperactive-impulsive symptoms and catastrophizing when upset and inattentive symptoms and distractibility when upset to later symptoms of depression. In addition, network comparisons demonstrated that higher polygenic risk for ADHD is associated with greater network connectivity and differences in network structure, particularly among ADHD symptoms and ER domains. Future research should consider catastrophizing and distractibility when upset as treatment targets to prevent the development of depression symptoms among early adolescents with ADHD symptoms.

## Supplementary Material

Supplementary Files

This is a list of supplementary files associated with this preprint. Click to download.

- SupplementaryInformation.docx

## Figures and Tables

**Table 1:** Baseline Demographic Characteristics of Overall Sample.

Demographic Variable		Full Sample		Year 4		Chi-squared
		*n*	%	*n*	%	*p*
Sex	Male	6,187	52.1	2,454	52.5	.837
	Female	5,676	47.8	2,223	47.5	
	Other	3	0.03	1	0.02	
Race/Ethnicity	White	6,172	52.0	2,652	56.7	< .001
	Black	1,783	15.0	503	10.8	
	Asian	252	2.1	108	2.3	
	Other	1,247	10.5	461	9.9	
	Hispanic/Latinx	2,410	20.3	954	20.4	.874
Language	English	11,215	94.5	4,406	94.2	.221
	Spanish	651	5.5	272	5.8	
Income	<$25,000	1,634	13.8	515	11.0	< .001
	$25,000–$49,999	1,588	13.4	630	13.5	
	$50,000–$99,999	3,068	25.9	1,280	27.4	
	$100,000– $199,999	3,310	27.9	1,394	29.8	
	$200,000+	1,250	10.5	516	11.0	
	Refused/Don’t Know	1,015	8.6	343	7.3	
Parent/Caregiver	Biological Mother	10,125	85.3	4,011	85.7	.140
	Biological Father	1,182	10.0	469	10.0	
	Other	559	4.7	198	4.2	
Parental Education	12^th^ grade or less (no high school diploma)	786	6.6	251	5.4	< .001
	High school or GED	1,260	10.6	410	8.8	
	Some college/Associate degree	3,481	29.3	1,329	28.4	
	Bachelor’s Degree	3,330	28.1	1,425	30.5	
	Graduate Degree	2,992	25.2	1,256	26.9	
	Refused	17	0.1	7	0.2	

*Note*. Demographic characteristics are reported by parents or caregivers at baseline, shown for the full sample of participants (*n* = 11,866) and for the subset of participants with year 4 data available (*n* = 4,678). Categories of race/ethnicity were mutually exclusive, and participants who identified as Hispanic or Latinx were not also counted under another category. Language indicates whether caregiver completed surveys in English or Spanish. Income refers to combined parental income of all parent(s). Chi-squared tests of independence compared whether demographic variables were associated with year 4 follow-up data availability.

**Table 2:** Descriptive Statistics of Nodes in the Network

Node	Timepoint	*n*	*M*	*SD*	median	range	*se*
CBCL ADHD Items							
4. Fails to finish things he/she starts	Average	11,866	0.5	0.5	0.3	2	<0.1
	Baseline	11,862	0.5	0.6	0	2	<0.1
	Year 1	11,201	0.5	0.6	0	2	<0.1
	Year 2	10,898	0.5	0.6	0	2	<0.1
8. Can’t concentrate, can’t pay attention for long	Average	11,866	0.5	0.6	0.3	2	<0.1
	Baseline	11,862	0.5	0.7	0	2	<0.1
	Year 1	11,201	0.4	0.6	0	2	<0.1
	Year 2	10,898	0.4	0.6	0	2	<0.1
10. Can’t sit still, restless, or hyperactive	Average	11,866	0.3	0.5	0	2	<0.1
	Baseline	11,862	0.4	0.6	0	2	<0.1
	Year 1	11,201	0.3	0.6	0	2	<0.1
	Year 2	10,898	0.3	0.5	0	2	<0.1
41. Impulsive or acts without thinking	Average	11,866	0.3	0.5	0	2	<0.1
	Baseline	11,861	0.3	0.5	0	2	<0.1
	Year 1	11,201	0.3	0.5	0	2	<0.1
	Year 2	10,897	0.3	0.5	0	2	<0.1
78. Inattentive or easily distracted	Average	11,866	0.5	0.6	0.3	2	<0.1
	Baseline	11,861	0.5	0.7	0	2	<0.1
	Year 1	11,201	0.5	0.7	0	2	<0.1
	Year 2	10,897	0.5	0.6	0	2	<0.1
93. Talks too much	Average	11,866	0.3	0.5	0	2	<0.1
	Baseline	11,861	0.3	0.5	0	2	<0.1
	Year 1	11,201	0.3	0.5	0	2	<0.1
	Year 2	10,897	0.3	0.5	0	2	<0.1
104. Unusually loud	Average	11,866	0.14	0.3	0	2	<0.1
	Baseline	11,861	0.15	0.4	0	2	<0.1
	Year 1	11,201	0.14	0.4	0	2	<0.1
	Year 2	10,897	0.13	0.4	0	2	<0.1
DERS-P Subscales							
Attuned	Year 3	9,939	0.9	0.4	0.8	1.7	<0.1
Catastrophize	Year 3	9,848	0.9	0.4	0.9	2.0	<0.1
Neg. Sec. Emotions	Year 3	9,854	0.9	0.4	0.8	2.3	<0.1
Distract	Year 3	9,965	0.9	0.4	0.8	1.6	<0.1
CBCL Depression Items							
Node	Timepoint	*n*	*M*	*SD*	median	range	*se*
14. Cries a lot	Year 4	4,678	0.1	0.3	0	2	<0.1
24. Doesn’t eat well	Year 4	4,678	0.3	0.5	0	2	<0.1
35. Feels worthless or inferior	Year 4	4,678	0.2	0.4	0	2	<0.1
52. Feels too guilty	Year 4	4,678	0.1	0.3	0	2	<0.1
54. Overtired without good reason	Year 4	4,677	0.1	0.4	0	2	<0.1
76. Sleeps less than most kids	Year 4	4,678	0.1	0.4	0	2	<0.1
77. Sleeps more than most kids during day/night	Year 4	4,678	0.1	0.3	0	2	<0.1
100. Trouble sleeping	Year 4	4,678	0.2	0.5	0	2	<0.1
102. Underactive, slow moving, or lacks energy	Year 4	4,678	0.2	0.4	0	2	<0.1
103. Unhappy, sad, or depressed	Year 4	4,678	0.2	0.5	0	2	<0.1

*Note. M* = mean, *SD* = standard deviation, *se* = standard error, CBCL = Child Behavior Checklist, DERS-P = Difficulties with Emotion Regulation Scale – Parent Report. DERS-P scores are standardized.

**Table 3:** Bootstrapped Strength, Expected Influence, and Bridge Expected Influence Estimates.

Node	Community	Strength	EI	Bridge EI
1. Finish_things	ADHD	1.01	0.84	0.28
2. Concentrate	ADHD	1.22	1.17	0.01
3. Hyperactive	ADHD	1.24	0.88	0.04
4. Impulsive	ADHD	1.11	0.86	0.11
5. Inattentive	ADHD	1.46	1.14	0.06
6. Talks_much	ADHD	1.15	0.65	0.02
7. Too_loud	ADHD	0.93	0.71	0.09
8. Enjoys_little	Depression	1.51	1.00	0.13
9. Cries_lot	Depression	1.31	0.60	−0.00
10. Eats_well	Depression	0.93	0.65	0.10
11. Worthless	Depression	1.42	1.20	0.23
12. Too_guilty	Depression	1.40	0.74	0.16
13. Overtired	Depression	1.52	1.30	−0.00
14. Less_sleep	Depression	1.51	0.82	0.14
15. More_sleep	Depression	1.46	0.74	0.10
16. Trouble_sleep	Depression	1.75	0.98	−0.06
17. Underactive	Depression	1.49	0.84	0.06
18. Unhappy	Depression	1.79	1.18	−0.10
19. Attuned	ER	0.96	0.20	0.10
20. Catastrophize	ER	1.64	1.40	0.28
21. Neg_Sec	ER	1.32	0.45	0.02
22. Distract	ER	1.15	0.86	0.29

*Note*. EI = Expected Influence, Neg_Sec = Negative Secondary Emotions, ER = Emotion regulation. Values are unstandardized bootstrapped estimates.

**Table 4:** Significant Network Comparisons by ADHD Polygenic Risk Score (PRS).

Node A	Node B	High PRS Network Edge	Low PRS Network Edge	Test statistic	*p*-value
Hyperactive	Impulsive	.22	.06	.16	.013
Concentrate	Inattentive	.61	.49	.12	.013
Impulsive	Inattentive	.16	.03	.12	.013
Hyperactive	Talks too much	.11	.00	.11	.013
Hyperactive	Too loud	.17	.00	.17	.013
Talks too much	Too loud	.35	.13	.22	.013
Catastrophize	Distract	.52	.40	.11	.013
Node	Centrality Measure	High PRS Value	Low PRS Value	Test statistic	*p*-value
Finish things	Strength	0.84	0.58	0.25	.004
	Expected Influence	0.84	0.58	0.25	.004
Concentrate	Strength	1.13	0.84	0.29	.004
	Expected Influence	1.13	0.84	0.29	.004
Hyperactive	Strength	0.99	0.37	0.62	.004
	Expected Influence	0.83	0.37	0.46	.004
Impulsive	Strength	0.83	0.41	0.43	.004
	Expected Influence	0.83	0.41	0.43	.004
Inattentive	Strength	1.21	0.75	0.46	.004
	Expected Influence	1.09	0.75	0.34	.004
Talks too much	Strength	0.79	0.13	0.66	.004
	Expected Influence	0.55	0.13	0.43	.004
Too loud	Strength	0.84	0.20	0.64	.004
Node	Centrality Measure	High PRS Value	Low PRS Value	Test statistic	*p*-value
Too loud	Expected Influence	0.84	0.20	0.64	.004
	Bridge Expected Influence	0.21	0.00	0.21	.016
Sleeps less	Expected Influence	0.62	0.40	0.22	.021
Attuned	Expected Influence	0.22	0.52	−0.29	.004
Catastrophize	Strength	1.34	1.13	0.21	.007
	Expected Influence	1.34	1.13	0.21	.004

*Note*. Networks were split above and below the sample median ADHD PRS. Centrality values are unstandardized. Edge weights are partial correlations and were refitted prior to estimation to increase precision. Only significant results are shown, *p* < .05.

## Data Availability

The datasets analyzed in the current study are stored by the NIMH Data Archive (https://nda.nih.gov/study.html?id=2313). Data can be accessed with approval through the NDA Data Use Certification.

## Footnotes

1 Centrality and bridge centrality results did not change in sensitivity analyses including adolescent self-reported depression symptoms, only complete cases, only English-speakers, and adding the two removed DERS-P items. Results using item-level DERS-P data were also examined. See Table S3 in supplementary information for results.

2 Sensitivity analyses of network comparison tests found a consistent pattern of omnibus test results across all three comparisons when using adolescent self-reported instead of parent-reported depression items. Some differences were found in individual centrality and edge invariance tests, which may be more sensitive to the fewer number of depression items available for adolescent self-report than parent-report. See Figures S6 and S7 in supplementary materials for visualization of results.

## List of Abbreviations

ADHD: attention deficit/hyperactivity disorder
ER: emotion regulation
ABCD Study: Adolescent Brain and Cognitive Development Study
DERS: Difficulties with Emotion Regulation Scale
DERS-P: Difficulties with Emotion Regulation Scale – Parent Report
CBCL: Child Behavior Checklist
PRS: polygenic risk score
GWAS: Genome-Wide Association Study
SNP: single nucleotide polymorphism
CS-coefficient: centrality stability coefficient
